# PCB Coil Pairs for Small Magnetic Volumes

**DOI:** 10.3390/nano16130801

**Published:** 2026-06-28

**Authors:** Howard R. Selden, Rebecca Y. Lai, Ryan A. Riskowski

**Affiliations:** 1 Department of Physics, University of Nebraska at Omaha, 6001 Dodge Street, Omaha, NE 68182, USA; howardselden@unomaha.edu; 2 Department of Chemistry, University of Nebraska Lincoln, 552 Hamilton Hall 639 N 12 Street, Lincoln, NE 68588, USA; rlai2@unl.edu

**Keywords:** magnetic nanoparticles, magnetic heating, radiofrequency heating, iron oxide nanoparticles

## Abstract

Here we describe a compact radiofrequency (RF) magnetic field generator designed to generate fields within millimeter-scale volumes. The device consists of printed multilayer circuit board (PCB) coils stacked in a paired parallel configuration. Compared to conventional solenoidal systems, this architecture significantly reduces device size, sample volume, and power requirements. We characterize the RF response of the system, including impedance and scattering parameters, and describe the behavior near ~330 kHz, which overlaps with frequencies common in magnetic nanoparticle heating, magnetic actuation, and other applications requiring localized fields. Electromagnetic modeling is used to evaluate magnetic field amplitude and spatial homogeneity (finding a maximum local deviation from the mean field of 5.5%). The mean B_z_ field across a 5 mm × 5 mm ROI centered between paired PCBs was 6.3 ± 0.1 mT/A (compared to 6.1 ± 1.0 mT/A measured experimentally). These results demonstrate operating parameters consistent with nanoparticle heating applications for PCB-based coil pairs.

## 1. Introduction

Decades of research into localized magnetic field generation has been motivated by nanoparticle hyperthermia for applications such as cancer treatment and drug delivery [1,2,3], as well as more recent efforts to remotely control biochemical processes and molecular systems [4,5,6]. Magnetic heating of nanoparticles is achieved through the application of alternating magnetic fields, where energy from the applied field is absorbed by the particle and dissipated as heat into the surrounding medium [7,8,9]. Because magnetic fields are not strongly attenuated by dielectric materials such as biological tissue, this approach enables selective heating of magnetic nanoparticles with minimal direct interaction with surrounding materials.

Standard systems used to generate RF alternating magnetic fields for heating of magnetic nanoparticles and related applications typically rely on macroscopic solenoidal coils that enclose centimeter-scale volumes (hundreds of cubic centimeters). Obtaining sufficient B-field amplitudes (~mT) at frequencies typically employed for magnetic heating (~100 kHz–1 MHz) is commonly achieved through bulky, power-intensive systems that are not easily adapted for spatially targeted, small-scale, or portable applications [4,10,11,12]. For emerging use cases such as microscale biochemical control, neural stimulation, or spatially resolved actuation, there is a need for compact field-generating devices capable of operating over much smaller volumes [4,11,13]. Table 1 shows a small cross-section of commercial and custom-made systems for induction heating of particles and other applications. Commercially available systems are reliable but use larger working volumes requiring kW powers. And while smaller, handmade systems can achieve reduced working volumes, they lack the reproducibility of commercial systems or manufactured PCBs.

**Figure 1:**
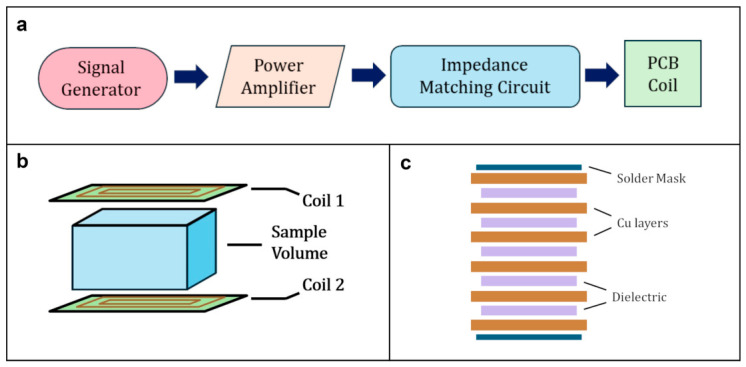
(**a**) Schematic of driving electronics for field generation. (**b**) Cartoon showing mm scale of volume accommodated by the coils. (**c**) Exploded view of PCBs depicting 6 copper layers of spiral trace per PCB.

Here we report the design and characterization of a miniaturized magnetic field generator based on printed circuit board (PCB) coils stacked in a Helmholtz-like configuration (Figure 1). By leveraging multilayer PCB fabrication, each coil consists of multiple stacked spiral traces connected in series, enabling high effective turn density within a compact footprint. While direct measurement of particle types and heating profiles are outside of the scope of this manuscript, the resulting device produces fields at ~6 mT per amp in volumes on the order of ~50 mm^3^, representing a reduction of several orders of magnitude compared to conventional systems for induction heating. These values fall well within the typical operating parameters of particle heating, actuation, and localized stimulation applications [1,2,4,5,7,8,9,18,19,20].

## 2. Materials and Methods

### 2.1. PCB Coil Fabrication

PCB coils were designed using KiCad PCB editor version 9.0 and physical prototypes produced by JLCPCB. Each coil consists of 6, 2 oz copper layers separated by FR-4 dielectric to make a total board thickness of ~1.79 mm. Each metal layer is composed of 9 right-handed square spirals, with each layer connected in series using vias. Two identical PCB coils were connected in parallel and stacked in a Helmholtz-like configuration. See the Supplementary Materials for design specifics.

### 2.2. Electronic Property Modeling

**Figure 2:**
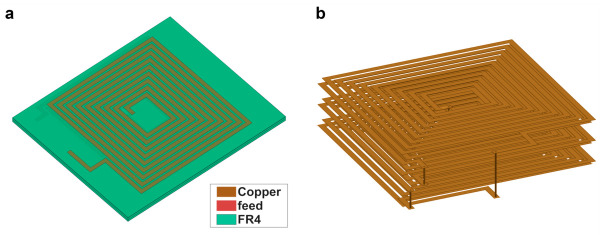
(**a**) Simulation model of coil pairs generated in this work for calculating coil parameters and magnetic fields. In simulations and experiments, identical PCB coils are held 2 mm apart. (**b**) Extracted copper traces from the PCB. Each PCB coil consists of 6 copper layers of square spiral trace with each layer connected in series by internal vias.

Electromagnetic simulations were performed using MatLab with associated RF PCB and Antenna toolboxes. Coil models were generated by directly importing the Gerber files produced with KiCad and used for PCB production (Figure 2a), with slight modification to the length of the input trace to accommodate MatLab feed points (Figure 2b). The coil geometry was discretized using a triangular surface mesh. Mesh density was selected to adequately resolve the trace geometry and current distribution, with convergence verified by refining the mesh until changes in the computed electronic properties were negligible. Values for impedance and s-parameters were computed directly with a method-of-moments-based solver appropriate for conductive structures at radiofrequencies. See the Supplementary Materials for model details and scripts.

### 2.3. Magnetic Field Modeling

Magnetic field simulations were performed using custom Mathematica scripts and a simplified filament-based model of traces. The coil geometry was discretized by generating filaments across the trace width and then weighting the value of each filament by the number of filaments (*n* = 3 here). A wire model of the square planar spiral was generated, and magnetic fields were computed using a Biot–Savart-based method [21,22]. Multilayer geometry of each PCB, and the stacked arrangement of two PCBs, was simulated by translating copies of a single simulated layer to appropriate z-heights according to physical layout. This model sidesteps voltage and impedance considerations by ignoring the mutual coupling between copper layers and between stacked boards, providing a more direct calculation of the field dependance on current present in the traces. See the Supplementary Materials for model details and scripts.

### 2.4. Experimental Circuit

**Figure 3:**
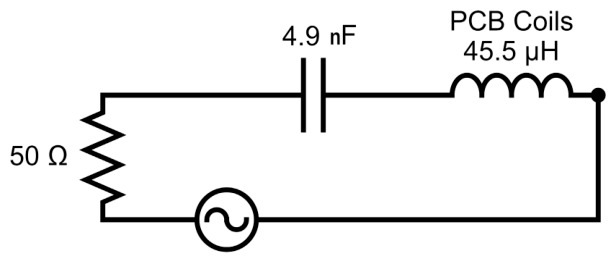
Diagram of test circuit containing the PCB coil stack. A capacitor is placed in series with the lumped PCB coil assembly (two stacked PCBs connected in parallel). A 50 ohm resistor is then placed in series to approximate load matching with the AC source and provide voltage measurements.

Determination of current in the circuit was performed by placing the inductive coils in series with a resonant capacitor and a resistor of known value (50 Ohms) and then measuring the voltage across the resistor to calculate current in the circuit (Figure 3). Resonance with the inductive coils was achieved with a handmade bank of five ~1 nF capacitors connected in parallel to achieve a measured 4.9 nF lumped capacitance. The 4.9 nF capacitor bank is connected in series with the parallel-connected PCB (45.5 µH) coil assembly to achieve resonance at ~330 kHz. A 50 Ohm resistor is used to approximate load matching with a Siglent SSG3021X function generator producing a 1 V (peak to peak) sine wave with a 50 Ohm output impedance. Current measurements were then made across the resistor using a Siglent SDS1000X-E oscilloscope during experiments.

### 2.5. Characterization Setup and Measurement of Bz

**Figure 4:**
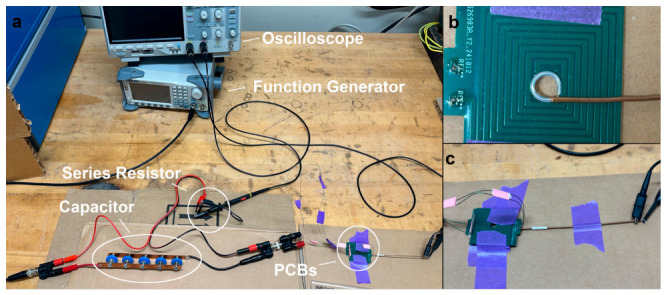
(**a**) Characterization setup including driving function generator, measurement oscilloscope, and the experimental circuit. The oscilloscope is connected across the series resistor to monitor circuit voltage and determine current, and is also connected to the pickup loop to measure induced emf and determine average field strength. (**b**) The pickup loop is sized and placed to collect the field measurement at the central region of interest for the PCB coils. (**c**) The pickup loop is placed between two PCB coils and affixed in place during measurement.

The characterization setup (Figure 4) includes the assembled circuit driven by a Siglent SSG3021X function generator producing a 1 V (peak to peak) sine wave at a 50 Ohm output impedance. Voltage measurements were then made across the resistor using a Siglent SDS1000X-E oscilloscope during experiments. Fields were measured using uninsulated coaxial wire formed into a single pickup loop of radius 3 mm. This loop was then directly affixed between two PCB coils and the spacing measured to be 2 mm. The benchtop function generator was used under constant voltage mode so that we could more cleanly find the circuit resonant frequency. Resonance is found by adjusting the frequency until the voltage drop across the series resistor maximizes, approaching very close to the voltage output of the function generator, and with the remaining voltage divided across the losses in the circuit (coils, capacitor, and connections).

## 3. Results

### 3.1. PCB Design and Electronic Properties

To reduce the size and power requirements of magnetic field generation, we sought to minimize the working magnetic volume, employing small, multilayer PCB coils stacked one above the other in a complementary Helmholtz-like configuration. The opposing faces of the coils are held 2 mm apart, and an area of 5 mm × 5 mm lying equidistant between the coils (1 mm) was chosen for the region of magnetic interest. This design greatly reduces the working volumes from cubic centimeters to cubic millimeters (~50 mm^3^). But inherent electrical properties such as inductance and impedance can greatly affect the power efficiency of field generation at relevant frequencies, and so must be examined for their impact on power requirements.

Each multilayer PCB consists of six 2 oz layers (~70 µm thick) of copper traces (1 mm wide) in square-planar spirals (0.5 mm pitch). The final design contains 9 loops per layer for a total of 54 loops per PCB, which we found to yield desirable electronic properties while maintaining manufacturability and mechanical stability. Using 2 oz copper layers with a trace width of 1 mm it is plausible to operate continuously up to 2 amps according to IPC-2152 guidelines. This multilayer approach allows for a much higher number of effective turns and B-field within a compact footprint but comes with the cost of increased inductance and AC impedance, requiring higher voltages to drive meaningful currents.

**Figure 5:**
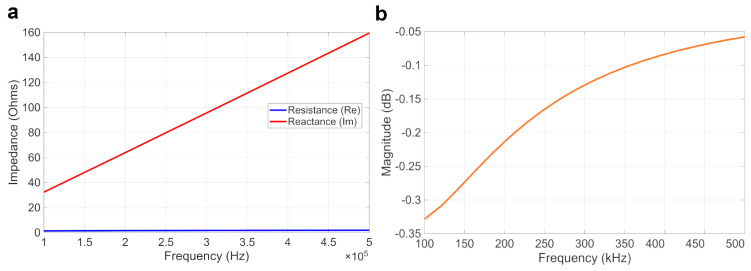
(**a**) Plot of impedance for a PCB coil from 100 to 500 kHz. Reactance (red) rises monotonically as a function of frequency and resistance (blue) decreases slightly with an average value of ~1.8 Ω. (**b**) Plot of S_11_ from 100 to 500 kHz showing the rise in reflected power as frequency increases toward MHz ranges.

Modeling of the PCB electronic properties was performed in MatLab using geometry imported directly from files containing the 3D model used for board production with slight post modification to extend the input traces. Using a method-of-moments approach that incorporates interlayer interactions, the impedance and effective inductance were calculated as a function of frequency about our circuit resonance of 330 kHz. The calculated impedance for a single PCB at 330 kHz is 1.8 + i105 Ω (Figure 5a) and Q value (Q = Im(Z)/Re(Z)) is ~58. The effective inductance (L_eff_ = Im(Z)/ω) was calculated by finding the mean value from 100 to 500 kHz and was found to be 50.9 ± 0.3 µH. This aligns with the measured inductance of 55.2 µH and supports the accuracy of the computational model used here. Reflection coefficients (S_11_) at the input of the PCB were determined for an individual board over the frequency range of 100–500 kHz (Figure 5b). The S_11_ values show that reflected power for the PCB is minimum near 100 kHz and increases with frequency, trending with the rising coil impedance. By placing the coils in series with a capacitor, it is possible to shift the frequency at which reflected power is minimal and current maximized, such as in our experimental setup which creates a reflected power minimum at 330 kHz.

### 3.2. Coil Simulation

When two PCB coils are in a stacked configuration, their fields will be in phase and add together. If the coils are separated by a distance equal to the coil radius in a Helmholtz configuration, the homogeneity of the magnetic field is maximized between stacked coils and in the sample volume. In this work, we reduced the separation distance between the stacked PCBs to 2 mm to achieve higher field strengths, meaning that local gradients will reduce uniformity of the magnetic field in the central region. Thus, it is important to model the PCB to examine the field for spatial homogeneity [11,23,24,25,26].

Magnetic field calculations of the PCB coils follow directly from the Biot–Savart law, with field amplitude scaling linearly with current in the low-frequency regime. Electrical and power limitations are primarily determined by resistive losses in the copper traces and the efficiency of impedance matching between the coils and the power source.

**Figure 6:**
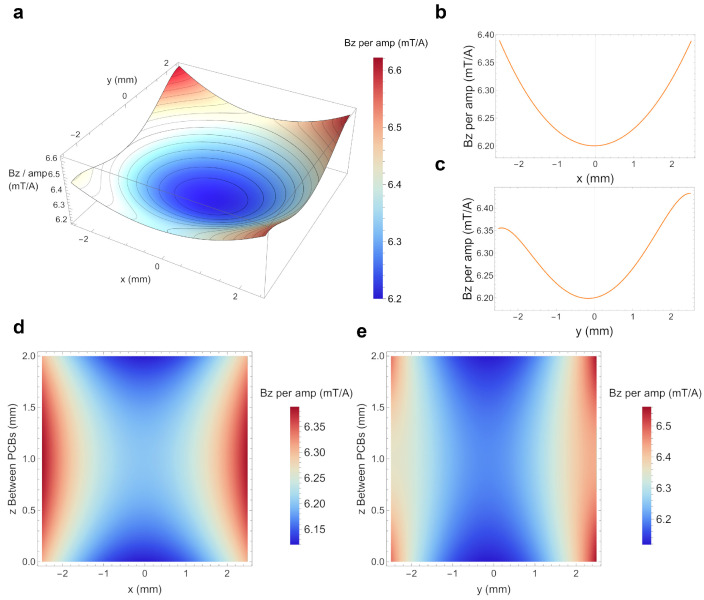
(**a**) Magnetic field strength per amp (|B_Z_|/amp) in the 5 mm × 5 mm region of interest between stacked PCBs and at the center of the spirals. The regions of increased field intensity at the edges of the region are due to the proximity of vias with high current density coming from the top and bottom PCBs. B_Z_ ranges ~+/− 0.2 mT/A across the region of interest giving a maximum deviation from the mean of 5.5%. (**b**,**c**) Cross-sections of |B_Z_|/amp along the x and y axes. (**d**,**e**) Heat maps of x–z at y = 0 and of y–z at x = 0.

Simulation of the coil fields was performed in Mathematica and the field profiles of single PCBs containing 6 metal layers and 54 total loops were overlapped to map the combined fields between the stacked coils. The simulated magnetic field distribution is shown in Figure 6, illustrating both the field amplitude and spatial homogeneity in the central 5 mm × 5 mm region of interest centered between the stacked PCB coils. The field strength across the xy plane of the central region of interest ranges from 6.2 to 6.4 mT per amp in the coil, with a mean of 6.3 mT ± 0.1 mT/A and a minimum value of 6.2 mT/A located at the center point (furthest from the current carrying traces). These results demonstrate that the coil device produces a well-defined magnetic field that is localized to a volume on the order of 50 mm^3^ (considering the full separation distance between the PCB coils). Comparing modeling and measurement as detailed below, we found that a 2 mm separation distance between PCBs (compared to an average coil radius of ~6.5 mm) provided strong field densities with relatively nominal change to field homogeneity. Using (B(x,y,z) − B_mean_)/B_mean_ across ~40,000 unique points in the volume, we find a maximum local deviation of 5.5% from the mean B-field amplitude. Figure 6a shows Bz across a 5 mm × 5 mm region of interest located at the midplane between two PCBs (z = 1 mm) where our pickup loop is placed during measurement (see Figure 4).

### 3.3. Coil Measurements

The measured inductance of single PCB coils was 56 µH compared to 54 µH calculated from the simulated PCB. The measured impedance was Z_m_ = 4.5 + *i*69 Ω at 200 kHz (limit of our LCR meter), while the simulated coils yielded Z_s_ = 1.6 + i64 Ω at 200 kHz, and the simulated impedance at our operating frequency of 330 kHz yields Z_s_ = 1.8 + i107 Ω, providing good confidence in the simulated coil properties despite the variation in real resistance due to surface roughness, connection losses, and test-fixture parasitics. The impedance measured at 200 kHz for the parallel-connected PCBs was 5.5 + i57 Ω. The inductance of two stacked PCB coils when connected in parallel was measured to be 46 µH, yielding a mutual inductance of 37 µH and a calculated impedance of Z_s_ = 3.6 + i95 Ω at 330 kHz.

To drive the PCB coil stack at the expected impedance for the power source (50 Ω), the coil stack was first placed in series with a capacitor bank to provide resonance and present minimal resistance and power losses. Using a handmade bank of high-voltage capacitors to create 4.9 nF, and with a measured inductance of 46 µH for the stacked coils connected in parallel, we found the calculated resonant frequency to be 335 kHz. This frequency was selected because it is lies well within the common frequency range used for inductive heating of Fe_3_O_4_ magnetic nanoparticles [7,27]. The observed resonance of the experimental circuit was found to be 330 kHz due to additional small stray capacitance and inductances. To monitor the current in the circuit and across the coils, a 50 Ohm resistor was added in series to the capacitor-coil element to approximate load matching with the source impedance.

**Figure 7:**
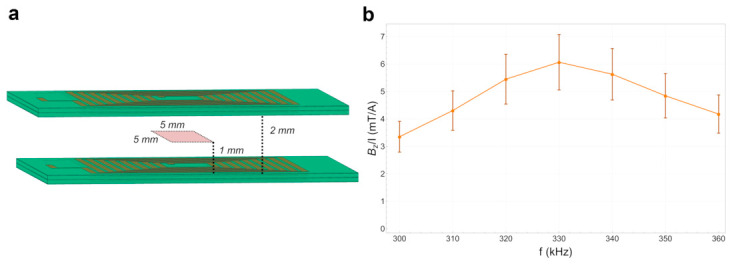
(**a**) Design of stacked PCB coils held 2 mm apart indicating the 5 mm × 5 mm ROI at the midplane. (**b**) Measured Bz in the ROI as a function of frequency around the circuit resonance. The decrease in efficiency away from the resonance frequency is due to increased power losses in the circuit.

The magnetic field between the coils was measured directly using a small length of uninsulated coaxial wire formed into a single pickup loop of radius 3 mm. The pickup loop was affixed between the stacked PCB coils at the region of interest (Figure 7a), and the voltage in the pickup loop was measured by an oscilloscope. The voltage measured at the oscilloscope is directly proportional to the magnitude of the average *B_z_* through the loop as seen in Equation (1).

(1) $$B_{z}=\frac{V_{Max}}{2\pi^{2}fr^{2}}\ldots$$

where *B_Max_* is the average magnetic field amplitude in the area of interest, *V_Max_* is the amplitude of the voltage measured in the pickup loop, *f* is the frequency of applied current to the coil, and *r* is the radius of the pickup loop. See the Supplementary Materials for full derivation and raw values.

For a figure of merit, and to give context to other coils and systems, the magnetic field strength can be expressed as the strength of the magnetic flux density per amp of current in the coil (*B_z_/I*). Here we found that the magnetic field maximized near the theoretical resonance of the experimental circuit where the power losses were minimal, yielding 6.1 ± 1.0 mT/A (Figure 7b). This measured value matches closely with the mean B-field values from simulation of 6.3 ± 0.1 mT/A.

The PCB coil traces can sustain currents of up to 2 A according to ICP-2152 guidelines with appropriate thermal management to produce B_z_ fields of ~12 mT under continuous operation. Comparing this value to applications like those found in Table 1 and reported in the literature, we find experimental values reported as low as 1.2 mT [28] and more commonly near ~30 mT, but achieved at kW powers [4,5,6,29,30,31,32,33,34]. Using a 50 Ω load matching condition, we find that assuming minimal losses our PCB coils only require powers on the order of ~10^2^ W to obtain these field strengths, representing an order-of-magnitude reduction in required power compared to larger solenoid systems.

Currents approaching 2A would require a little over 200 W of rms power across our circuit and, with a measured real resistance of 5.5 Ω, the power dissipated across the coils and their connections will be 12.2 W—6.6% of the applied power and fraction of the total measured circuit resistance of 84 Ω. Operation of the coils at these currents would require considerable thermal mitigation due to the compact multilayer structure. In addition to external cooling solutions such as air or liquid flow, through-board vias can be added to increase heat exchange from internal layers. Thermal improvements will be a subject of future work on this field-generating approach.

For further context, a common figure of merit is the time-averaged off-target power dissipation in the surrounding medium given by *H*_0_ × *f*. This value is often associated with off-target tissue heating in biological samples and a safe physiological limit is given as *H*_0_ × *f* ≤ 5 × 10^9^ Am^−1^ s^−1^, where *H*_0_ is the maximum magnetic field in A/m (H = B/µ_0_) at the applied frequency *f* [35]. Using the maximum field values given above of ~12 mT at 330 kHz, we find *H*_0_ × *f* = 3 × 10^9^ Am^−1^ s^−1^ and within the upper bounds of physiological limits.

## 4. Conclusions

We designed and tested a PCB coil pair and measured B-field strengths and electrical properties compatible with applications requiring strong localized fields such as inductive heating, magnetic actuation, and stimulation. Within the 5 mm × 5 mm ROI, the measured mean B_z_ was as high as 6.1 ± 1.0 mT per current amp in the coil at a frequency of 330 kHz—well within the range of reported frequencies and field strengths typical of inductive particle heating. Computational modeling showed good field homogeneity with a mean B_z_ of 6.3 ± 0.1 mT/A, a coefficient of variation (RMS nonuniformity) of 1.4% and a maximum local deviation of 5.5% from the mean. This platform dramatically reduces the size of inductive heating devices and increases special control of particle heating applications by confining the magnetic volume to a reduced mm scale (~50 mm^3^). By increasing the spatial control over magnetic particle heating, it is possible to adapt this technique to multistage platforms such as ‘lab on a chip’ devices or molecular sensors, broadening the overall toolbox of nanoparticle applications.

## Figures and Tables

**Table 1:** Some field-generating systems for small magnetic volumes.

System	Coil Type	Frequency	Field	Working Volume	Power	Reference
UltraFlex, N-Series	Solenoid	323 kHz	~47 mT	~100 cm^3^	kW	[14]
Ambrell, HotShot	Solenoid	386 kHz	~7 mT	~100 cm^3^	kW	[15]
Nanotherics, Magnetherm	Solenoid	109 kHz	~20 mT	~100 cm^3^	kW	[16]
Hand-wrapped ferrite toroid	Gapped Toroid	~0.1–1 MHz	~20–80 mT	~2 cm^3^	~10^2^ W	[10]
Handmade	Solenoid	~500 kHz	~10 mT	~50 mm^3^	kW	[17]
*This paper* PCB coils	Square Planar	~330 kHz	~6 mT/Amp	~50 mm^3^	~10^2^ W	This paper

## Data Availability

Measurement data available in Supplementary Materials.

## Supplementary Materials

The following supporting information can be downloaded at: https://www.mdpi.com/article/10.3390/nano16130801/s1, Figure S1: PCB design details; Figure S2: coil trace geometry used in Biot-Savart calculation; Table S1: measured values of magnetic field [21].
