# Supplementary material for: PCB Coil Pairs for Small Magnetic Volumes
Source: Nanomaterials (Basel). 2026 Jun 28;16(13):801. doi: 10.3390/nano16130801 (PMC13363308; doi:10.3390/nano16130801)
Supplement: Supplementary file 1 [file nanomaterials-16-00801-s001.zip › nanomaterials-4364679-supplementary.pdf]

Supporting Information:

PCB Coil Pairs for Small Magnetic Volumes

Howard R Selden [1], Rebecca Y. Lai [2], Ryan A. Riskowski [1,*]

[1] Department of Physics, University of Nebraska at Omaha, 6001 Dodge Street Omaha, NE 68182
[2] Department of Chemistry, University of Nebraska Lincoln, 552 Hamilton Hall 639 N 12 Street Lincoln, NE 68588
* Author to whom correspondence should be addressed: rriskowski@nebraska.edu

Table of Contents

S1..........................PCB Design Parameters
S2..........................Calculation of B fields using Biot-Savart Law
S3..........................Derivation of Relation Between Measured Voltage and B_z
Appendix A............Mathematica Script for Field Calculations
Appendix B............MatLab Script for Electronic Properties

S1. PCB Design Parameters

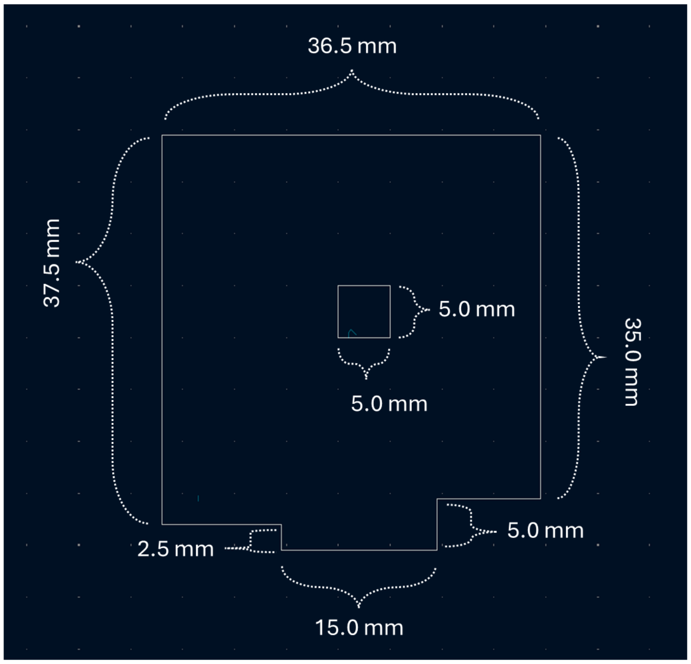

PCB Layer Thickness (μm)	
Copper L_1	70
Dielectric (FR4)	100
Copper L_2	70
Dielectric (FR4)	535
Copper L_3	70
Dielectric (FR4)	100
Copper L_4	70
Dielectric (FR4)	535
Copper L_5	70
Dielectric (FR4)	100
Copper L_6	70

Copper weight: 2 oz
Trace thickness: 1 mm
Trace pitch: 0.5 mm
Spirals per layer: 9
Via Diameter: 0.5 mm
Via through-hole: 0.3 mm

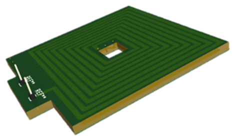

Figure S1. Design parameters of the manufactured PCBs used in this work.

S2. Calculation of B fields using Biot-Savart

We used a simulation proposed by Tian et al. that calculates the magnetic flux density for square planar spiral coils. The simulation uses the Biot-Savart law for a finite length wire to calculate the magnetic flux density in the *z*-direction for each trace. Using a square approximation of the geometry of the coil and the Biot-Savart solution, where *I* represents the current at some time, *r'* represents a position vector of some point along a trace, *dl(r')* represents taking the integral along the path of the current, and *r* represents the position vector of the calculation, and *z'* represents the *z*-position of the trace (adapted from Tian et al., 2022)[1]

$$dB = \frac{\mu_0}{4\pi} I(t) \oint \frac{dl(r') \times (r' - r)}{|r - r'|^3}$$

The solution of this, as provided by Tian et al. produces a set of equations that outputs vector values for the magnetic flux density at a position in *x*, *y*, and *z*, with trace length *a*. B_{AB}, B_{BC}, B_{CD}, and B_{DA} represent the factors produced by the integration that produce one of the components when multiplied by its partial derivative in that direction.

$$\vec{B}_{AB} = \frac{\mu_0 I}{4\pi\sqrt{\left(\frac{a}{2}+y\right)^2 + z^2}} \cdot \left(\frac{\frac{a}{2}+x}{\sqrt{\left(\frac{a}{2}+x\right)^2 + \left(\frac{a}{2}+y\right)^2 + z^2}} + \frac{\frac{a}{2}-x}{\sqrt{\left(\frac{a}{2}-x\right)^2 + \left(\frac{a}{2}+y\right)^2 + z^2}} \right)$$

$$\vec{B}_{BC} = \frac{\mu_0 I}{4\pi\sqrt{\left(\frac{a}{2}-x\right)^2 + z^2}} \cdot \left(\frac{\frac{a}{2}+y}{\sqrt{\left(\frac{a}{2}-x\right)^2 + \left(\frac{a}{2}+y\right)^2 + z^2}} + \frac{\frac{a}{2}-y}{\sqrt{\left(\frac{a}{2}-x\right)^2 + \left(\frac{a}{2}-y\right)^2 + z^2}} \right)$$

$$\vec{B}_{CD} = \frac{\mu_0 I}{4\pi\sqrt{\left(\frac{a}{2}-y\right)^2 + z^2}} \cdot \left(\frac{\frac{a}{2}-x}{\sqrt{\left(\frac{a}{2}-x\right)^2 + \left(\frac{a}{2}-y\right)^2 + z^2}} + \frac{\frac{a}{2}+x}{\sqrt{\left(\frac{a}{2}+x\right)^2 + \left(\frac{a}{2}-y\right)^2 + z^2}} \right)$$

$$\vec{B}_{DA} = \frac{\mu_0 I}{4\pi\sqrt{\left(\frac{a}{2}+x\right)^2 + z^2}} \cdot \left(\frac{\frac{a}{2}-y}{\sqrt{\left(\frac{a}{2}+x\right)^2 + \left(\frac{a}{2}-y\right)^2 + z^2}} + \frac{\frac{a}{2}+y}{\sqrt{\left(\frac{a}{2}+x\right)^2 + \left(\frac{a}{2}+y\right)^2 + z^2}} \right)$$

These factors are then multiplied by their partials with respect to the *z*-direction and summed to output the *z*-component of the magnetic flux density:

$$\vec{B}_z = \sum \left(B_{AB} \cdot \frac{\frac{a}{2} + y}{\sqrt{\left(\frac{a}{2} + y\right)^2 + z^2}} \right) + \sum \left(B_{BC} \cdot \frac{\frac{a}{2} - x}{\sqrt{\left(\frac{a}{2} - x\right)^2 + z^2}} \right)$$

$$+ \sum \left(B_{CD} \cdot \frac{\frac{a}{2} - y}{\sqrt{\left(\frac{a}{2} - y\right)^2 + z^2}} \right) + \sum \left(B_{DA} \cdot \frac{\frac{a}{2} + x}{\sqrt{\left(\frac{a}{2} + x\right)^2 + z^2}} \right)$$

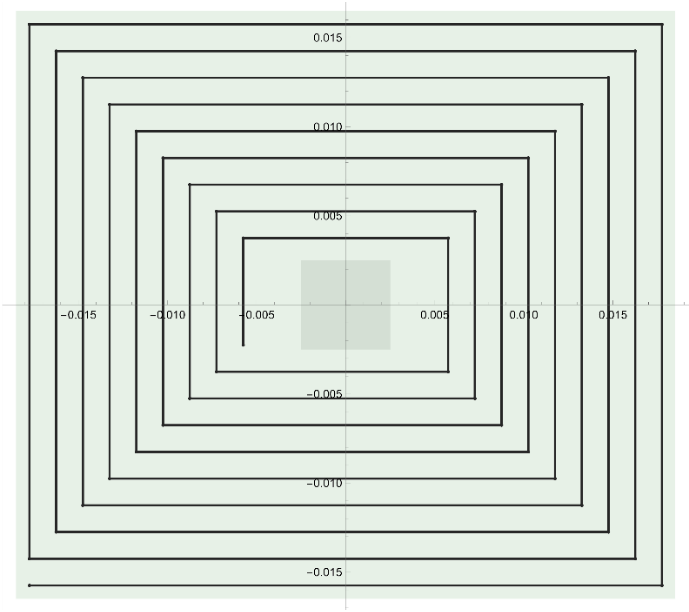

Figure S2. Diagram of segmented square spiral used to simulate each layer of a PCB and implemented in Mathematica to calculate the field at our ROI.

S3. Derivation of Relation Between Measured Voltage and B_z

Given an alternating current, the current as a function of time can be written, where *i* represents the current at an instant, *I* represents the maximum current, *f* represents the frequency, and *t* represents a point in time:

$$i = I \sin(2\pi f t)$$

Using Ohm's law and assuming that the resistance of the circuit outside the resistor is negligible, the function can be rewritten, where V_R represents the maximum voltage across the resistor, *R* is resistance of the resistor:

$$i = \frac{V_R}{R} \sin(2\pi f t)$$

Since the magnetic flux density through the loop is proportional to the current in the circuit, the function for magnetic flux in the loop at some time can be described, where *B* represents the magnetic flux density at an instant and $B_{z\text{-}max}$ represents the maximum magnetic flux density orthogonal to the loop:

$$B_z = B_{z-max} \sin(2\pi f t)$$

Due to Faraday's Law, the function for induced voltage in the loop at an instant can be written, where v_{loop} represents the voltage across the loop and *A* represents the area of the loop:

$$v_{loop} = -A \frac{dB_z}{dt}$$

$$\frac{dB_z}{dt} = -2\pi f B_{z-max} \cos(2\pi f t)$$

Since the derivative of the magnetic flux density and the voltage across the loop must be proportional to each other, they must both be cosine functions. Therefore, the voltage across the loop at an instant can be expressed where V_{loop} represents the maximum voltage across the loop:

$$v_{loop} = -V_{loop} \cos(2\pi f t)$$

$$V_{loop} = 2\pi A f B_{z-max}$$

$$B_{z-max} = \frac{V_{loop}}{2\pi f A}$$

Since the area of the loop approximates a circle, the expression is written where *r* represents the radius of the loop:

$$B_{z-max} = \frac{V_{loop}}{2\pi^2 f r^2}$$

Uncertainty was calculated using propagation of error, where σ_B, $\sigma_{V\text{-}loop}$, and σ_r represent the uncertainties in the magnetic flux density, and the measurements of the voltage across the loop and the radius of the loop. The uncertainty of the voltage of the loop was ± 0.040 mV in the scaling used in the trials. The uncertainty of the measurement of the radius of the loop was ± 0.500 mm. The uncertainty of the frequency was negligible:

$$\sigma_B = \sqrt{\left(\frac{\partial B_{z-max}}{\partial V_{loop}} \sigma_{V-loop}\right)^2 + \left(\frac{\partial B_{z-max}}{\partial r} \sigma_r\right)^2}$$

After calculating this, the term that comes from the uncertainty in the measurement of the voltage across the loop is negligible when compared to the term that comes from the uncertainty of the measurement of the radius of the loop, which is orders of magnitude larger in all of our trials:

$$\sigma_B = \frac{1}{f}\sqrt{\left(0.225\ \frac{V^2}{m^4}\right) + (8.80 * 10^5\ m^{-4})V_{loop}^2}$$

Table S1. Measured Values of Bz/Current

f (kHz)	B_z/I (mT/A)	Error
300	3.4	0.6
310	4.3	0.7
320	5.4	0.9
330	6.1	1.0
340	5.6	0.9
350	4.8	0.8
360	4.2	0.7

References

1. Tian, L., Song, L., Zheng, Y. & Wang, J. A 4 × 4 array multichannel magnetic stimulation system using submillimeter sized planar square spiral coils: The spatial distribution of electromagnetic field. *International Journal of Applied Electromagnetics and Mechanics* **68**, 329–346 (2022).

Appendix A. Mathematica Script for Field Calculations

```
(****************************************************)
(* === Initial Parameters === *)
(****************************************************)

(* Geometry (mm) *)
outerXmm=37;   (*X dimension of outer rectangle*)
outerYmm=33.0;   (*Y dimension of outer rectangle*)
innerXmm=5;    (*X dimension of inner clearance/target*)
innerYmm=5;    (*Y dimension of inner clearance/target*)
wmm=1.0;   (*trace width*)
smm=0.5;   (*edge-to-edge spacing between traces*)
centerStepmm=wmm+smm;  (*centerline step per half-turn inward*)
thickmm=0.07;  (*copper thickness in mm (~70 μm) *)

(* Current& constants *)
Iamp=1.0;      (* current amplitude for B/I *)
mu0=4 Pi 10^-7; (*Magnetic constant*)

(* Filament bundle resolution *)
nW=3;  (*filaments across width *)
nT=1;  (*filaments across thickness*)

(* ==== Plot grid (mm)==== *)
xSpanmm=5;ySpanmm=5;              (*mid-plane window to visualize*)
nx=121;ny=121;       (*grid resolution*)
zPlotmm=0.0;              (*mid-plane for this layer*)

(* Convert mm to m *)
mmToM[x_]:=x*10^-3;

(*Unit vector*)
UnitV[v_]:=v/Norm[v];

(*For a segment in a layer that lies in the XY plane*)
LocalFrame[a_,b_]:=Module[{t,z={0.,0.,1.},n},t=UnitV[b-a];(*along segment*)n=UnitV[Cross[z,t]];(*in-plane
normal*){t,n,z}];

(****************************************************)
(* === Function to Build Sprial Geometry === *)
(****************************************************)
RectSpiralPath[outerX_,outerY_,innerX_,innerY_,centerStep_,z0_:0.,maxHalfTurns_:100,cwQ_:True]:=Module
[{hx=outerX/2.,hy=outerY/2.,step=centerStep,xL,xR,yB,yT,pts={},k=0,canContinue=True,canAddLeftLeg},(*cen
terline "bounding box" after accounting initial half-step to center*)
  xL=-hx+step/2;
  xR=+hx-step/2;
  yB=-hy+step/2;
  yT=+hy-step/2;
  AppendTo[pts,{xL,yB,z0}];
```

```
 While[canContinue&&k<maxHalfTurns,
  (*bottom edge->right edge->top edge->left edge (one rectangular ring)*)
  AppendTo[pts,{xR,yB,z0}];
  k++;
  If[k>=maxHalfTurns,Break[]];
  AppendTo[pts,{xR,yT,z0}];
  k++;
  If[k>=maxHalfTurns,Break[]];
  AppendTo[pts,{xL,yT,z0}];
  k++;
  If[k>=maxHalfTurns,Break[]];

  (*step inward for next half-turn start*)

  xR-=step;
  yB+=step;
  yT-=step;

  (*stop if continuing would trespass the inner clearance on next segment*)
  canAddLeftLeg=Abs[xL]>innerX/2;
  If[canAddLeftLeg,AppendTo[pts,{xL,yB,z0}];
   xL+=step;
   k++;
   If[k>=maxHalfTurns,Break[];],Null];
  canContinue=canAddLeftLeg&&(Abs[yB]>innerY/2)&&(xR-xL>innerX);
  ];
 If[!cwQ,pts=Reverse[pts]];
 pts];

(*****************************************************)
(* === Make Polylines for Filament Bundle === *)
(*****************************************************)
PolylineToSegments[path_List]:=Partition[path,2,1];

(*Build offset filaments across width/thickness;weights sum to 1*)
FilamentizeSegment[{a_,b_},width_,thick_,nW_:3,nT_:1,wW_:Automatic,wT_:Automatic]:=Module[{t,n,z,alpha
s,betas,wWi,wTj,weights,offs},{t,n,z}=LocalFrame[a,b];
 alphas=Subdivide[-1,1,nW-1];(*across width*)betas=If[nT==1,{0.},Subdivide[-1,1,nT-
1]];(*thickness*)wWi=If[wW===Automatic,ConstantArray[1./nW,nW],wW/Total[wW]];
 wTj=If[wT===Automatic,ConstantArray[1./nT,nT],wT/Total[wT]];
 weights=Flatten[Outer[Times,wWi,wTj]];
 offs=Flatten[Table[(width/2) alphas[[i]] n+(thick/2) betas[[j]] z,{i,nW},{j,nT}],1];
 MapThread[{a+#1,b+#1,#2}&,{offs,weights}]];

(*****************************************************)
(* === Biot Savart Law ==== *)
(*****************************************************)
SegmentB[r_,a_,b_,I_]:=Module[{ra,rb,la,lb,ell,num,den},
 ra=r-a;rb=r-b;
 la=Norm[ra];
 lb=Norm[rb];
 ell=b-a;
 num=Cross[ell,ra/la+rb/lb];
```

```
  den=la*lb+ra.rb;(* =la*lb+(r-a).(r-b)*)
  (mu0 I/(4 Pi))*(num/den)];

CoilB[r_,filSegs_List,I_:1.]:=Total[SegmentB[r,#[[1]],#[[2]],I*#[[3]]]&/@filSegs];

(*****************************************************)
(* === Build a Layer ==== *)
(*****************************************************)
(*Convert mm to m*)
outerX=mmToM[outerXmm];outerY=mmToM[outerYmm];
innerX=mmToM[innerXmm];innerY=mmToM[innerYmm];
w=mmToM[wmm];s=mmToM[smm];
centerStep=mmToM[centerStepmm];
thick=mmToM[thickmm];

z0=mmToM[zPlotmm];
layerZOffsets=mmToM/@{0.,-0.17,-0.775,-0.945,-1.55,-1.72};
pcbObservationOffset=mmToM[1.];
zAbovePCB=z0+pcbObservationOffset;
zBelowPCB=z0+Min[layerZOffsets]-pcbObservationOffset;

(*Generate centerline for one layer*)
paths3D=RectSpiralPath[outerX,outerY,innerX,innerY,centerStep,z0+#,37 (*maxHalfTurns*),True
(*CW*)]&/@layerZOffsets;
path1=First[paths3D];

(*Segments*)
segs1=PolylineToSegments[path1];
segsByLayer=PolylineToSegments/@paths3D;

(*Filamentize all segments*)
filSegs1=Flatten[FilamentizeSegment[#,w,thick,nW,nT]&/@Flatten[segsByLayer,1],1];
TwoPCBFieldAt[x_,y_]:=CoilB[{x,y,zAbovePCB},filSegs1,Iamp]+CoilB[{x,y,zBelowPCB},filSegs1,Iamp];

(*****************************************************)
(* === Calcumalate Fields=== *)
(*****************************************************)
(*Center Field Check*)
Bcenter=TwoPCBFieldAt[0.,0.];
BzPerAmp=Bcenter[[3]]/Iamp//N
BzPermTPerAmp=1000 BzPerAmp

(*Midplane 2D slice of Bz*)

xVals=Subdivide[-mmToM[xSpanmm]/2,mmToM[xSpanmm]/2,nx-1];
yVals=Subdivide[-mmToM[ySpanmm]/2,mmToM[ySpanmm]/2,ny-1];
xValsmm=1000 xVals;
yValsmm=1000 yVals;

bzGrid=Table[TwoPCBFieldAt[x,y][[3]]/Iamp,(*Bz / amp*){y,yVals},{x,xVals}];
bzGridmTPerAmp=1000 bzGrid;
```

```
FlotPlot=ListDensityPlot[bzGridmTPerAmp,DataRange->{{xValsmm[[1]],xValsmm[[-
1]]},{yValsmm[[1]],yValsmm[[-1]]}},FrameLabel->{Style["x (mm)",16,Black],Style["y
(mm)",16,Black]},FrameStyle->Directive[Black,14],LabelStyle->Directive[Black,16],PlotLegends-
>BarLegend[Automatic,LegendLabel->Style["Bz per amp (mT/A)",16,Black],LabelStyle-
>Directive[Black,16]],InterpolationOrder->1,ColorFunction->"ThermometerColors"]

(*on-axis Y slice*)
bzAxis=Table[TwoPCBFieldAt[0.,y][[3]]/Iamp,{y,yVals}];
bzAxismTPerAmp=1000 bzAxis;

YZSlice=ListLinePlot[
 Transpose[{yValsmm,bzAxismTPerAmp}],Frame->True,FrameLabel->{"y (mm)","Bz per amp (mT/A)"},Prolog->
  {EdgeForm[],Orange,Opacity[0.1],Rectangle[{-1000 innerY/2,Min[bzAxismTPerAmp]},{1000
innerY/2,Max[bzAxismTPerAmp]}]}]


(****************************************************)
(* === Visualization === *)
(****************************************************)


SetOptions[ListLinePlot,LabelStyle->Directive[Black,FontSize->28],FrameTicksStyle->Directive[FontSize-
>20]];
(*on-axis X slice*)
bzXAxis=Table[TwoPCBFieldAt[x,0.][[3]]/Iamp,{x,xVals}];
bzXAxismTPerAmp=1000 bzXAxis;




XZSlice=ListLinePlot[
 Transpose[{xValsmm,bzXAxismTPerAmp}],
 Frame->True,FrameLabel->{"x (mm)","Bz per amp (mT/A)"},
 PlotRange->All,
 PlotStyle->{Orange, Thick},
 ImageSize->600,
 ImagePadding->{{100,40},{80,40}}
 ]

YZSlice2=ListLinePlot[
 Transpose[{yValsmm,bzAxismTPerAmp}],
 Frame->True,FrameLabel->{"y (mm)","Bz per amp (mT/A)"},
 BaseStyle->Directive[FontFamily->"Arial"],
 PlotStyle->{Orange,Thick},
 ImageSize->600,
 ImagePadding->{{100,40},{80,40}}
 ]

xyzPlot=ListPlot3D[
 bzGridmTPerAmp,
 DataRange->{{xValsmm[[1]],xValsmm[[-1]]},{yValsmm[[1]],yValsmm[[-1]]}},
 PlotRange->All,InterpolationOrder->2,
 AxesLabel->{
```

```
   Style["x (mm)",12],
   Style["y (mm)",12],
   Style[Column[{"Bz / amp","(mT/A)"}],12]
   },
  BoxRatios->{1,1,0.4},
  LabelStyle->Directive[Black,FontSize->12],
  TicksStyle->Directive[FontSize->10],
  BaseStyle->Directive[Black,FontFamily->"Arial"],
  ColorFunction->"ThermometerColors",
  MeshFunctions->{#3&},(*contours in height*)Mesh->15,MeshStyle->Opacity[0.35],
  ImageSize -> 450

  ]
(*Bz along the center z-axis between the two PCBs*)
zAxisN = 201;
interBoardGap = 2 pcbObservationOffset;
lowerBoardTopSurface = zAbovePCB - pcbObservationOffset;
upperBoardBottomSurface = lowerBoardTopSurface + interBoardGap;
upperBoardShift =
  upperBoardBottomSurface - (zBelowPCB + pcbObservationOffset);
zAxisVals =
  Subdivide[lowerBoardTopSurface, upperBoardBottomSurface, zAxisN - 1];
zGapValsmm = 1000 (zAxisVals - lowerBoardTopSurface);

TwoPCBCenterFieldAtZ[z_]:=CoilB[{0.,0.,z},filSegs1,Iamp]+CoilB[{0.,0.,z-upperBoardShift},filSegs1,Iamp];
bzZAxis=Table[TwoPCBCenterFieldAtZ[z][[3]]/Iamp,{z,zAxisVals}];
bzZAxismTPerAmp=1000 bzZAxis;

ZAxisSlice = ListLinePlot[
  Transpose[{zGapValsmm, bzZAxismTPerAmp}],
  Frame -> True,
  FrameLabel -> {"z Between PCBs (mm)", "Bz per amp (mT/A)"},
  PlotRange -> All,
  PlotStyle -> {Blue, Thick},
  ImageSize -> 600,
  ImagePadding -> {{100, 40}, {80, 40}}
  ]

(* Bz x-z heatmap at y=0 between the two PCBs*)
TwoPCBFieldAtXZ[x_, z_] :=
  CoilB[{x, 0., z}, filSegs1, Iamp] +
   CoilB[{x, 0., z - upperBoardShift}, filSegs1, Iamp];
bzXZGrid =
  Table[TwoPCBFieldAtXZ[x, z][[3]]/Iamp, {z, zAxisVals}, {x, xVals}];
bzXZGridmTPerAmp = 1000 bzXZGrid;

XZHeatmap = ListDensityPlot[
  bzXZGridmTPerAmp,
  DataRange -> {{xValsmm[[1]], xValsmm[[-1]]}, {zGapValsmm[[1]],
    zGapValsmm[[-1]]}},
  FrameLabel -> {Style["x (mm)", 16, Black],
   Style["z Between PCBs (mm)", 16, Black]},
  FrameStyle -> Directive[Black, 14],
```

```
  LabelStyle -> Directive[Black, 16],
  PlotLegends ->
   BarLegend[Automatic,
    LegendLabel -> Style["Bz per amp (mT/A)", 16, Black],
    LabelStyle -> Directive[Black, 16]], InterpolationOrder -> 1,
  ColorFunction -> "ThermometerColors"
  ]

(*Bz y-z heatmap at x=0 between the two PCBs*)
TwoPCBFieldAtYZ[y_, z_] :=
  CoilB[{0., y, z}, filSegs1, Iamp] +
   CoilB[{0., y, z - upperBoardShift}, filSegs1, Iamp];
bzYZGrid =
  Table[TwoPCBFieldAtYZ[y, z][[3]]/Iamp, {z, zAxisVals}, {y, yVals}];
bzYZGridmTPerAmp = 1000 bzYZGrid;

YZHeatmap =
 ListDensityPlot[bzYZGridmTPerAmp,
  DataRange -> {{yValsmm[[1]], yValsmm[[-1]]}, {zGapValsmm[[1]],
     zGapValsmm[[-1]]}},
  FrameLabel -> {Style["y (mm)", 16, Black],
    Style["z Between PCBs (mm)", 16, Black]},
  FrameStyle -> Directive[Black, 14],
  LabelStyle -> Directive[Black, 16],
  PlotLegends ->
   BarLegend[Automatic,
    LegendLabel -> Style["Bz per amp (mT/A)", 16, Black],
    LabelStyle -> Directive[Black, 16]], InterpolationOrder -> 1,
  ColorFunction -> "ThermometerColors"]




(* — — — Helpers — — — *)
(*Accepts either {x,y} or {x,y,z};returns {x,y}*)
XYOnly[path_List]:=path/. {x_?NumericQ,y_?NumericQ,___}:>{x,y};

(*Graphics for points+connecting segments,with optional numbering*)
Options[SpiralDebugPlot]={"ShowIndices"->True,(* "PointSize"->Medium, *)"LineThickness"-
>Thick,"AspectRatio"->Automatic,"Axes"->True,"PlotRange"->All};

SpiralDebugPlot[path_List,opts:OptionsPattern[]]:=Module[{xy=XYOnly[path],segs,pointLabels,showIdx,(* ps,
*) lt,ar,ax,pr},showIdx=OptionValue["ShowIndices"];
   (* ps=OptionValue["PointSize"]; *)
   lt=OptionValue["LineThickness"];
   ar=OptionValue["AspectRatio"];
   ax=OptionValue["Axes"];
   pr=OptionValue["PlotRange"];
   segs=Line@Partition[xy,2,1];
   pointLabels=If[showIdx,MapIndexed[With[{p=#1,i=First@#2},Text[Style[i,10,Black,Bold],p,{0,-1.2}]]&,xy],{}];
   Graphics[{Directive[GrayLevel[0.15],lt],segs,(* Directive[Red,ps], *) Point[xy],pointLabels},Axes-
>ax,AspectRatio->ar,PlotRange->pr]];
```

```
(*A quick report of segment lengths& directions *)
SpiralPathReport[path_List]:=Module[{xy=XYOnly[path],segs,vecs,lens,dirs},segs=Partition[xy,2,1];
  vecs=Subtract@@@segs;
  lens=Norm/@vecs;
  dirs=Normalize/@vecs;
  Dataset@MapIndexed[<|"Segment"->First@#2,"From"->segs[[First@#2,1]],"To"->segs[[First@#2,2]],"Δx"->vecs[[First@#2,1]],"Δy"->vecs[[First@#2,2]],"Length"->#1,"Dir"->dirs[[First@#2]]|>&,lens]];

path=RectSpiralPath[outerX,outerY,innerX,innerY,centerStep,0.0,38,True];

SpiralDebugPlot[path,"ShowIndices"->True]

Sketch
=With[{xy=XYOnly[path],ox=outerX,oy=outerY,ix=innerX,iy=innerY},Show[Graphics[{Darker[Green],Opacity[.2],Rectangle[{-ox/2,-oy/2},{ox/2,oy/2}],Directive[Gray,Dashed],Rectangle[{-ix/2,-iy/2},{ix/2,iy/2}]},Axes->True,AspectRatio->Automatic,PlotRange->All],SpiralDebugPlot[xy,"ShowIndices"->False]]]

(* Calculating mean and variance *)
flatB=Flatten[bzGridmTPerAmp];
meanB=Mean[flatB]
dev = StandardDeviation[flatB]
p2pPercent=100*(Max[flatB]-Min[flatB])/meanB
cvPercent=100*StandardDeviation[flatB]/meanB
maxAbsDevPercent=100*Max[Abs[flatB-meanB]]/meanB
```

Appendix B. MATLAB Script for Electronic Properties

```
% Layer stackup %
% ---Cu---     Layer{1}  [0.07 mm] *Feed Layer*
% ---FR4---    Layer{2}  [0.10 mm]
% ---Cu---     Layer{3}  [0.07 mm]
% ---FR4---    Layer{4}  [0.535mm]
% ---Cu---     Layer{5}  [0.07 mm]
% ---FR4------  Layer{6}  [0.10 mm]
% ---Cu---     Layer{7}  [0.07 mm]
% ---FR4---    Layer{8}  [0.535mm]
% ---Cu---     Layer{9}  [0.07 mm]
% ---FR4---    Layer{10} [0.10 mm]
% ---Cu---     Layer{11} [0.07 mm] *Feed Layer*

%% ------------------------------- %%
%  -------- Set up gerbers -------  %
%  ---------------------------------  %

% Set file path for gerber locations %
gerberFolder = "C:\..........";  %% Path Redacted

% Copper gerbers (top -> bottom)
```

```
gCu = { ...
  fullfile(gerberFolder,"6_layer_buildup_FlatEdges-F_Cu_ExtraBit.gtl") ...
  fullfile(gerberFolder,"6_layer_buildup_FlatEdges-In1_Cu.g1") ...
  fullfile(gerberFolder,"6_layer_buildup_FlatEdges-In2_Cu.g2") ...
  fullfile(gerberFolder,"6_layer_buildup_FlatEdges-In3_Cu.g3") ...
  fullfile(gerberFolder,"6_layer_buildup_FlatEdges-In4_Cu.g4") ...
  fullfile(gerberFolder,"6_layer_buildup_FlatEdges-B_Cu.gbl") ...
};

% ------- Set Some Initial Parameters ----- %

% Stack thicknesses (meters): 5 dielectric gaps between 6 copper layers
tGap = [ 0.10 0.535 0.10 0.535 0.10 ]*1e-3;

% Dielectric properties
er   = 4.2;
tanD = 0.02;

% Copper thickness (meters)
tCu = 70e-6;   % 0.07 mm = 70 µm


% ------- Pull shapes from gerbers------- %
% Read copper layers
CuShape = cell(1,6);
for k = 1:6
   s_Cu = gerberRead(gCu{k});   % returns geometry usable by pcbStack
   CuShape{k} = shapes(s_Cu);
end


% ================================= %%
%  ========= BUILD PCBSTACK =========  %
%  ================================  %
pcb = pcbStack;

% Create a BoardShape - using a simple rectangle
pcb.BoardShape = antenna.Rectangle( ...
'Length', 0.04, ...
'Width',  0.047, ...
'Center', [127.5e-3 -77.5e-3] ...
);

% --- BUILD DIELECTRICS ---
d = cell(1,5);
for k = 1:5
   d{k} = dielectric("FR4");
   d{k}.EpsilonR = er;
   d{k}.LossTangent = tanD;
   d{k}.Thickness = tGap(k);
end

% Conductor definition
```

```
m = metal("Copper");
m.Thickness = tCu;
pcb.Conductor = m;


% Set the Dielectric thickness summed up below the top copper.
pcb.BoardThickness = sum(tGap); % -- Board Thickness --%


% ======== Layer buildup ======== %
pcb.Layers = { ...
   CuShape{1}, ...
   d{1}, ...
   CuShape{2}, ...
   d{2}, ...
   CuShape{3}, ...
   d{3}, ...
   CuShape{4}, ...
   d{4}, ...
   CuShape{5}, ...
   d{5}, ...
   CuShape{6}, ...
};


% ------------------------------------ %
% -------- Feeds and Vias --------- %
% -------------------------------------%

% Feed locations (meters) from KiCad
pPlus  = [124e-3, -98e-3];
pMinus = [131e-3, -98e-3];

% point the feeds at copper layers
feedLayer = [1,11];

% assign Feed Locations to pcbStack
%pMinus(1) pMinus(2) feedLayer(1)];
pcb.FeedLocations = [ pPlus(1)  pPlus(2)  feedLayer(1) feedLayer(2)];
pcb.FeedDiameter = 0.25e-3; % Set feed diameter
pcb.FeedVoltage = 1;      % Set feed voltage

% Via Locations
pcb.ViaLocations = [...
   124e-3 -79.5e-3  1  3; ...    % cu_1--cu_2
   111.5e-3 -97e-3  3  5; ...    % cu_2--cu_3
   124e-3 -77.5e-3  5  7; ...    % cu_3--cu_4
   109e-3   -97e-3  7  9; ...    % cu_4--cu_5
   124e-3 -75.5e-3  9 11; ...    % cu_5--cu_6
   ];

% Set Via diameter
pcb.ViaDiameter = 0.25e-3;
```
